# Evaluation of the inhibition potential of plumbagin against cytochrome P450 using LC-MS/MS and cocktail approach

**DOI:** 10.1038/srep28482

**Published:** 2016-06-22

**Authors:** Ang Chen, Xiaojing Zhou, Shuowen Tang, Mingyao Liu, Xin Wang

**Affiliations:** 1 Shanghai Key Laboratory of Regulatory Biology, Institute of Biomedical Sciences and School of Life Sciences, East China Normal University, Shanghai, China; 2 Center for Translational Cancer Research, Institute of Biosciences and Technology, Texas A&M University Health Science Center, Houston, Texas, USA

## Abstract

Plumbagin (5-hydroxy-2-methyl-1,4-naphthoquinone), a natural naphthoquinone compound isolated from roots of *Plumbago zeylanica* L., has drawn a lot of attention for its plenty of pharmacological properties including antidiabetes and anti-cancer. The aim of this study was to investigate the effects of plumbagin on CYP1A2, CYP2B1/6, CYP2C9/11, CYP2D1/6, CYP2E1 and CYP3A2/4 activities in human and rat liver and evaluate the potential herb-drug interactions using the cocktail approach. All CYP substrates and their metabolites were analyzed using high-performance liquid chromatography–tandem mass spectrometry (LC-MS/MS). Plumbagin presented non-time-dependent inhibition of CYP activities in both human and rat liver. In humans, plumbagin was not only a mixed inhibitor of CYP2B6, CYP2C9, CYP2D6, CYP2E1 and CYP3A4, but also a non-competitive inhibitor of CYP1A2, with *K*_i_ values no more than 2.16 μM. In rats, the mixed inhibition of CYP1A2 and CYP2D1, and competitive inhibition for CYP2B1, CYP2C11 and CYP2E1 with *K*_i_ values less than 9.93 μM were observed. In general, the relatively low *K*_i_ values of plumbagin in humans would have a high potential to cause the toxicity and drug interactions involving CYP enzymes.

*Plumbago zeylanica* L. (family: Plumbaginaceae), also known as Chitrak, is a medicinal plant and mainly distributed around Africa and Asia including India and China^1^,^2^. It is originally documented in “Tang Ben Cao” in China and widely used in prescription for treating many diseases such as rheumatic joint pain, blood stasis amenorrhea and malignant sore in Traditional Chinese medicine^3^. Plumbagin (5-hydroxy-2-methyl-1,4-naphthoquinone, Fig. 1a) isolated from roots of *Plumbago zeylanica* L. is an active naphthoquinone and responsible for its therapeutic effects^4^. Previous studies have reported that plumbagin possesses lots of pharmacological properties including antidiabetic, anti-inflammatory, antibacterial and anti-immediate allergic reaction activities^1^,^5^. In particular, plumbagin presents significant anticancer activity^6^. For example, plumbagin can induce apoptosis and autophagy of human prostate cancer cells via sirtuin1- and PI3K/Akt/mTOR-mediated pathways^7^. And it also can kill human gastric cancer cells by induction of SH2-containing protein tyrosine phosphatase 1^8^. Therefore, Plumbagin is considered to be a promising antitumor candidate for drug development.

The cytochrome P450 (CYP) enzymes play an important role in metabolism of xenobiotics and endogenous substances. Almost 90% drugs are metabolized by CYP superfamily^9^,^10^. Among the CYP isoforms identified, the major human CYP isoforms (CYP1A2, CYP2B6, CYP2C9, CYP2D6, CYP2E1 and CYP3A4) are responsible for 80% of the CYP-mediated metabolism^11^,^12^,^13^. However, the inhibition and induction of CYP by herbs often occur, which is directly linked to herb-drug interactions (HDIs) and result in the toxicity and/or therapeutic failure^14^,^15^. In consequence, U.S. Food and Drug Administration (FDA) has recommended to use CYP-associated metabolic studies *in vitro* to predict the potential HDIs, which is the major attrition in drug development^16^. Recently, one study only found the inhibitory effects of plumbagin on CYP1A2, CYP2C19 and CYP3A4^17^. However, the mechanisms of CYP inhibition remain unclear. Further studies should be carried out to illustrate the mechanisms of plumbagin on CYP enzymes.

The aim of this study was to investigate the effects of plumbagin on several major CYP activities both in human and rat liver, including CYP1A2, CYP2B1/6, CYP2C9/11, CYP2D1/6, CYP2E1 and CYP3A2/4, and further explore the mechanisms of plumbagin on CYP inhibitory properties. In this report, the cocktail approach using classical substrates including phenacetin (CYP1A2), bupropion (CYP2B1/6), tolbutamide (CYP2C9/11), dextromethorphan (CYP2D1/6), chlorzoxazone (CYP2E1) and midazolam (CYP3A2/4)^16^, alternatively named the n-in-one assay, was adopted to monitor several CYP activities in a single experiment, which was much comprehensive, time and resource efficient^10^,^18^. In this approach, a liquid chromatography tandem mass spectrometry (LC-MS/MS) was usually used for the simultaneous detection of the formation of metabolites of these probe substrates to calculate the enzymatic reaction rates, which could be used to evaluate the corresponding CYP enzyme activities^19^. To our knowledge, this is the first study to report the potential effects of plumbagin on CYP2B1/6, CYP2C9/11, CYP2E1 and CYP2D1/6, and demonstrate the inhibitory mechanisms of CYPs. Therefore, the present study could be helpful to evaluate the potential HDIs involved with plumbagin comprehensively.

## Results

### Inhibition effects of plumbagin on CYP1A2, CYP2B6, CYP2C9, CYP2D6, CYP2E1 and CYP3A4 activities in human liver

**Figure 1:**
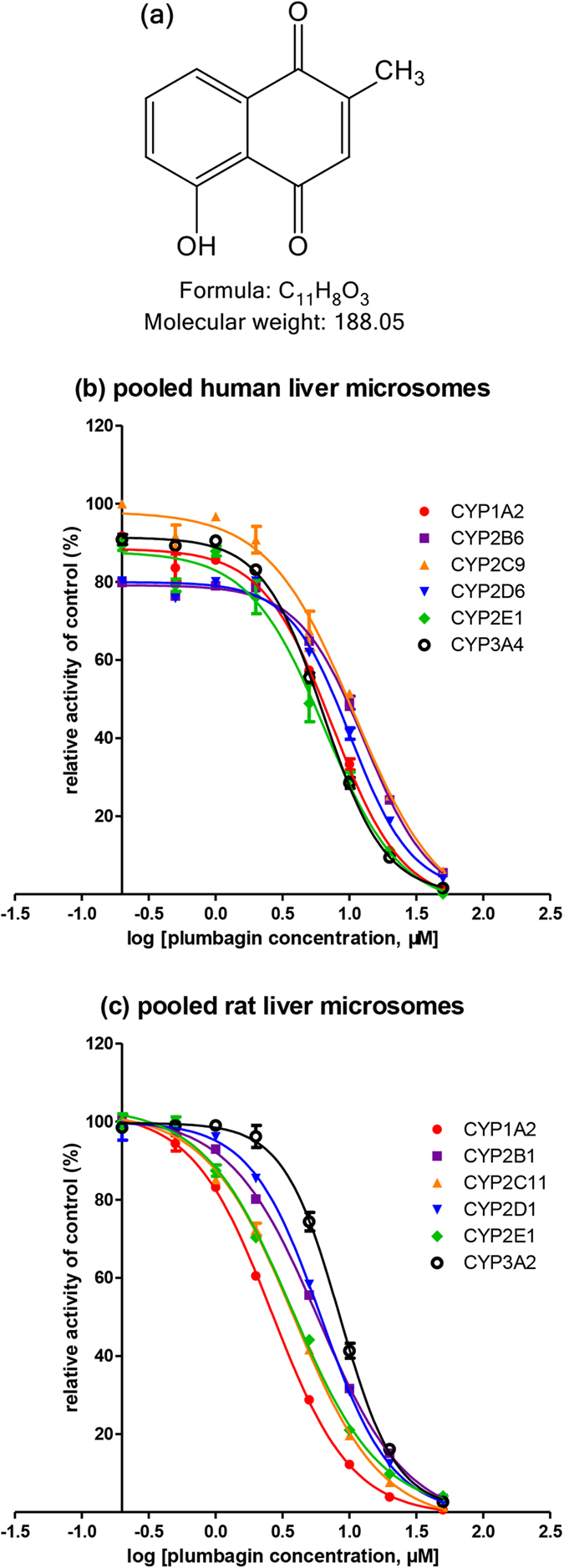
Chemical structure of plumbagin (a) and the inhibitory effects of CYPs activities by plumbagin in pooled human liver microsomes (b) and pooled rat liver microsomes (c). Chemical structure was produced using ChemDraw Professional 15.0. Results were mean ± SD of quadruplicate assays. The data was fit to log (plumbagin concentration) and normalized response equations using GraphPad Prism 5.0.

To investigate the effects of plumbagin on human CYP activities, various concentrations of plumbagin ranging from 0.05 μM to 50 μM were used to determine IC_50_ (Fig. 1b). Plumbagin inhibited CYP1A2-catalyzed phenacetin O-deethylation, CYP2B6-catalyzed bupropion hydroxylation, CYP2C9-catalyzed tolbutamide methyl-hydroxylation, CYP2D6-catalyzed dextromethorphan O-demethylation, CYP2E1-catalyzed chlorzoxazone 6-hydroxylation and CYP3A4-catalyzed midazolam 1-hydroxylation with IC_50_ values at 7.49 μM, 13.10 μM, 10.94 μM, 10.21 μM, 6.54 μM and 6.45 μM in HLMs, respectively (Table 1). These data certificated that plumbagin exhibited potently inhibitory effects on the major human six CYPs, including CYP1A2, CYP2B6, CYP2C9, CYP2D6, CYP2E1 and CYP3A4.

### Inhibition effects of plumbagin on CYP1A2, CYP2B1, CYP2C11, CYP2D1, CYP2E1 and CYP3A2 activities in rat liver

In rat liver, plumbagin also inhibited CYP1A2-catalyzed phenacetin O-deethylation, CYP2B1-catalyzed bupropion hydroxylation, CYP2C11-catalyzed tolbutamide methyl-hydroxylation, CYP2D1-catalyzed dextromethorphan O-demethylation, CYP2E1-catalyzed chlorzoxazone 6-hydroxylation and CYP3A2-catalyzed midazolam 1-hydroxylation with IC_50_ values at 2.65 μM, 5.86 μM, 3.85 μM, 6.17 μM, 3.72 μM and 8.47 μM, respectively (Table 1). In brief, plumbagin strongly inhibited all selected CYP-catalyzed probe reactions in a concentration-dependent manner (Fig. 1c), which agreed with the data of humans.

### Enzyme kinetic analysis for inhibition of CYPs by plumbagin in human liver

**Figure 2:**
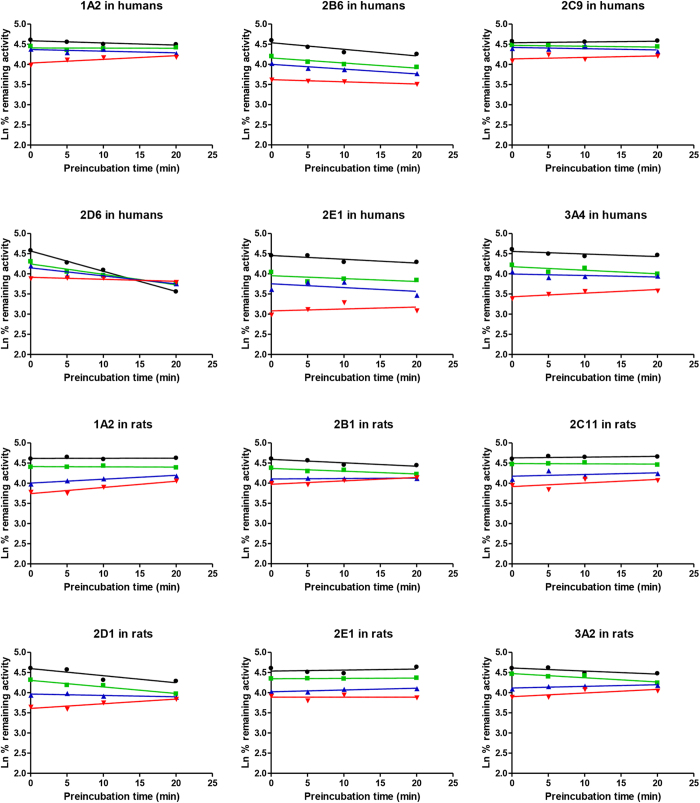
Time- and concentration-dependent inhibition of human and rat liver microsomal CYPs. The figures were prepared by a semilogarithmic plot of percent remaining activity *vs* incubation time. Microsomal reaction mixtures were pre-incubated for 20 min without (⚫) or with plumbagin at 0.5 μM (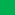), 1 μM (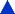), 2 μM (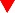) in humans or plumbagin at 0.2 μM (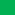), 0.5 μM (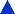), 1 μM (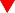) in rats. Each data point represents the mean of quadruplicate determinations.

To investigate whether the inhibition of CYPs by plumbagin was time-dependent, CYP activities were determined by pre-incubating microsomal incubation mixtures with NADPH for 0, 5, 10 and 20 min at 37 °C. Plumbagin showed no apparent time-dependent inhibition on all CYPs (CYP1A2, CYP2B6, CYP2C9, CYP2D6, CYP2E1 and CYP3A4) (Fig. 2).

**Figure 3:**
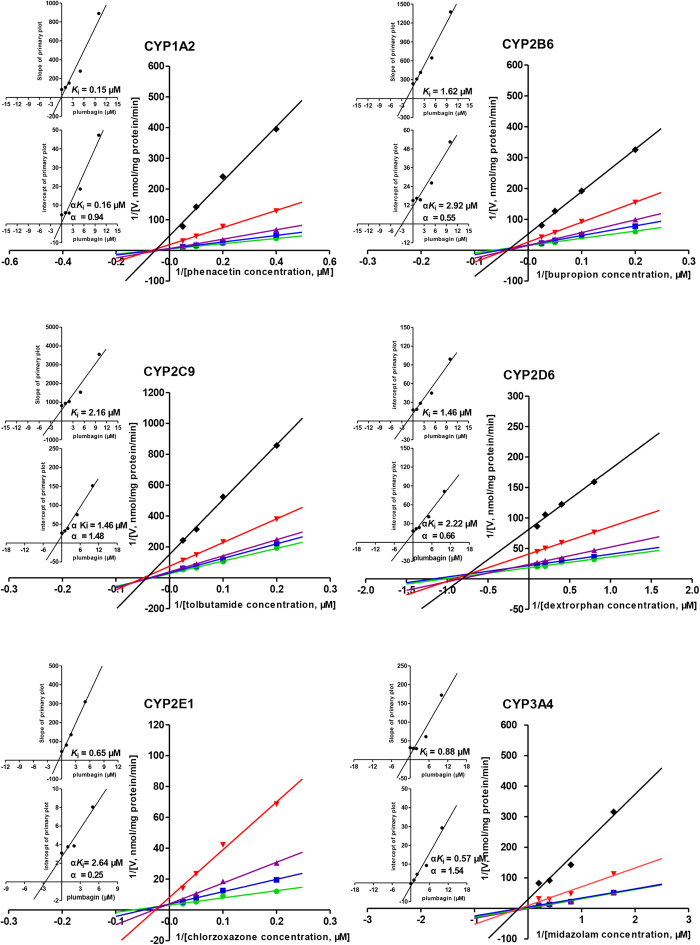
Primary Lineweaver–Burk plot, the secondary plot for *K*_i_ and the secondary plot for α*K*_i_ in the inhibition of CYP-mediated probe substrate metabolism by various concentrations of plumbagin (0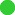, 1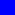, 2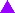, 5 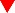, 10♦ μM) in pooled human liver microsomes. Phenacetin was used at concentrations of 2.5, 5, 10 and 20 μM; Bupropion was used at 5, 10, 20 and 40 μM; Tolbutamide was used at 5, 10, 20 and 40 μM; Dextromethorphan was used at 1.25, 2.5, 5 and 10 μM; Chlorzoxazone was used at 5, 10, 20 and 40 μM; Midazolam was used at 0.625, 1.25, 2.5 and 5 μM. Each data point represents the mean of quadruplicate determinations.

To further determine the types of CYP inhibition by plumbagin in HLMs, enzyme kinetics studies were carried out with various substrates concentrations in the presence or absence of plumbagin. The Lineweaver-Burk transformation of the enzyme velocities versus substrates concentrations showed that the types of inhibition by plumbagin for these selected CYPs were mixed inhibition on CYP2B6, CYP2C9, CYP2D6, CYP2E1 and CYP3A4, but non-competitive for CYP1A2. The *K*_i_ values were determined using the secondary Lineweaver–Burk plot, and then calculated at 0.15 μM, 1.62 μM, 2.16 μM, 1.46 μM, 0.65 μM and 0.88 μM for CYP1A2, CYP2B6, CYP2C9, CYP2D6, CYP2E1 and CYP3A4, respectively. From the secondary plot of Lineweaver–Burk plot for α*K*_i_, α*K*_i_ values were 0.16 μM, 2.92 μM, 1.46 μM, 2.22 μM, 2.64 μM and 0.57 μM for CYP1A2, CYP2B6, CYP2C9, CYP2D6, CYP2E1 and CYP3A4, respectively. Since α values of CYP2B6, CYP2C9, CYP2D6, CYP2E1 and CYP3A4 were not equal to 1, the types of inhibition by plumbagin for these five CYPs were mixed inhibition. However, the type of inhibition by plumbagin for CYP1A2 was non-competitive inhibition due to α value of CYP1A2 approximately equal to 1 (Table 1, Fig. 3).

### Enzyme kinetic analysis for inhibition of CYPs by plumbagin in rat liver

**Figure 4:**
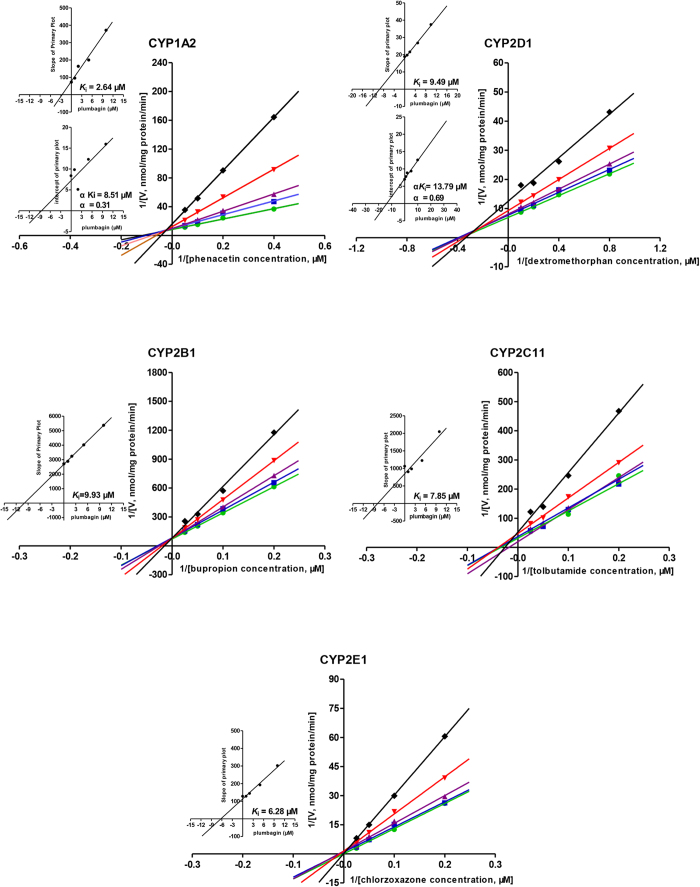
Primary Lineweaver–Burk plot, the secondary plot for *K*_i_ and the secondary plot for α*K*_i_ in the inhibition of CYP-mediated probe substrate metabolism by various concentrations of plumbagin (0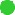, 1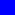, 2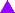, 5 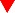, 10 ♦ μM) in rat liver microsomes. Phenacetin was used at concentrations of 2.5, 5, 10 and 20 μM; Bupropion was used at 5, 10, 20 and 40 μM; Tolbutamide was used at 5, 10, 20 and 40 μM; Dextromethorphan was used at 1.25, 2.5, 5 and 10 μM; Chlorzoxazone was used at 5, 10, 20 and 40 μM. Each data point represents the mean of six rats.

**Figure 5:**
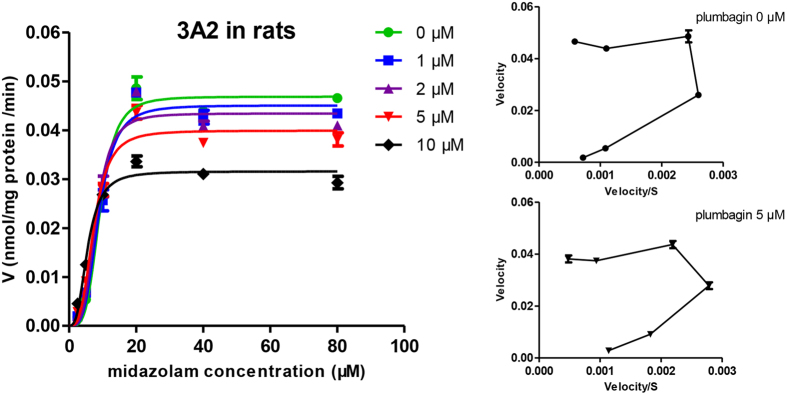
Sigmoidal auto-activation kinetics curve of CYP3A2 by various concentrations of plumbagin (0 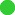, 1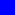, 2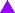, 5 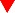, 10 ♦ μM) in rat liver microsomes. Midazolam was used at concentrations of 2.5, 5, 10, 20, 40 and 80 μM. Insets show the corresponding Eadie-Hofstee plots for CYP3A2 kinetic profile.

The time-dependent inhibition experiments were also performed and no time-dependent inhibition was observed in RLMs (Fig. 2). The Lineweaver-Burk transformation of the enzyme velocities versus substrates concentrations showed that the types of inhibition by plumbagin for these selected CYPs were mixed inhibition on CYP1A2 and CYP2D1, but competitive for CYP2B1, CYP2C11 and CYP2E1. The *K*_i_ values were calculated to be 2.64 μM, 9.93 μM, 7.85 μM, 9.49 μM and 6.28 μM for CYP1A2, CYP2B1, CYP2C11, CYP2D1 and CYP2E1, respectively. From the secondary plot of Lineweaver-Burk plot for α*K*_i_, α*K*_i_ values were 8.51 μM and 13.79 μM for CYP1A2 and CYP2D1, respectively. The types of inhibition by plumbagin on CYP1A2 and CYP2D1 were mixed inhibition, because α values of CYP1A2 and CYP2D1 were not equal to 01 (Table 1, Fig. 4). Moreover, the enzyme inhibition kinetics of CYP3A2 did not conform to the classical Michaelis–Menten kinetics (Fig. 5), which would be discussed in Discussion section.

## Discussion

*Plumbago zeylanica* L. has been widely used as traditional herbal medicine among many Chinese minorities for many years. Meanwhile, the medicinal plant is the main ingredient of Qubai Babuqi Pian (Sinopharm XinJiang Pharmaceutical Co., Ltd.) which is used to treat leucoderma in Uighur medicine. Nowadays, herbal medicines, especially being consumed along with modern drugs, are increasingly popular when patients are in several diseases or conditions^11^,^14^. When they are co-administered over overlapping time periods, the possibility of HDIs mediated by CYPs might exist^20^,^21^. However, the inhibition effects of plumbagin on CYP activities were rarely concerned. Among all CYP isoforms in humans, CYP3A4, CYP2C9, CYP1A2, CYP2E1, CYP2D6 and CYP2B6 are relative abundant, which accounts about for 40%, 20%, 13%, 10%, 2% and 2% in total CYPs respectively, and responsible for metabolism of the majority of clinically prescribed drugs^14^,^22^,^23^,^24^. CYP1A2 is involved in the metabolism of a variety of clinically using drugs, such as clozapine, tacrine, tizanidine, theophylline, and several important endogenous compounds^22^,^24^,^25^,^26^. CYP2B6 is responsible for approximately 3–8% of widely used pharmaceuticals and also reported to have high expression in breast tumors^23^,^27^,^28^. CYP2C9 contributes to the metabolism of not only commonly prescribed drugs which accounts for 10–20%, but also fatty acids, prostanoids and steroid hormones^22^. CYP2D6 expressing in liver and extrahepatic organs mediates approximately 25% of marketed drugs, such as antidepressants and antipsychotics^29^. Meanwhile, CYP2D6 with decreased activity is implicated in Parkinson disease in Caucasian populations^29^. CYP2E1 as a natural ethanol-inducible enzyme metabolically activates various carcinogens. Increased CYP2E1 activity is linked to the carcinogenic process^30^. Furthermore, more than 50% of drugs were metabolized by CYP3A4 including antibiotics, antiviral drugs, benzodiazepines, calcium channel blockers, statins and immunosuppressive drugs^31^,^32^. However, to our knowledge, the inhibition effects of plumbagin on these important CYPs have not been reported except for CYP1A2 and CYP3A4^17^. In particular, the mechanisms of inhibition of all six CYPs were completely investigated for the first time. The purpose of our work was to investigate the effects of plumbagin on the major CYP activities both in human and rat liver and assess the potential HDIs involved plumbagin or *Plumbago zeylanica* L.

To evaluate the potential HDIs, the cocktail approach, frequently used for CYP inhibition studies^33^,^34^,^35^, has been developed for monitoring CYP activities in a single run by LC-MS/MS with high sensitivity and selectivity. According to FDA guidance, classical probe substrates including phenacetin (CYP1A2), bupropion (CYP2B1/6), tolbutamide (CYP2C9/11), dextromethorphan (CYP2D1/6), chlorzoxazone (CYP2E1) and midazolam (CYP3A2/4) were selected. The concentrations of probe substrates were close to the *K*_m_ values of each CYP-catalyzed reaction according to published papers^16^,^33^. Given that the linear initial rates of metabolites formation for all selected CYPs with minimal substrates depletion and the automatic inactivation of CYPs in 37 °C comprehensively, the incubation time was chosen to 20 min. Moreover, the microsomal protein concentration in HLMs was 0.5 mg/ml, which was the most frequently employed, and high protein concentrations ought to be avoided because of the possibility of protein binding^18^.

To investigate whether inhibition of CYPs by plumbagin was time-dependent, time-dependent inhibition assays were performed with various plumbagin concentrations close to IC_25_, which was the most sensitive point for detecting a time-dependent inhibitor^36^,^37^. Too low concentrations of plumbagin may miss inactivation, but too high concentrations of plumbagin may cause much reversible inhibition to make the effect of time-dependent inhibition not obvious^36^. Meanwhile, considering not only automatic inactivation of some CYPs but also weak inhibitors which could not be identified within a short inactivation time, 20 min was used for this time-dependent inhibition assays. From Fig. 2, the activity of CYP2D6 in HLMs decreased slightly in a time-dependent manner without any inhibitor, which could be explained for easily automatic inactivation of CYP2D6 in HLMs. On the other hand, the inhibition effects on several CYPs by plumbagin were reduced with pre-incubation with NADPH, which could be due to metabolic depletion of plumbagin leading to diminished inhibitory capability^36^,^38^. Therefore, no time-dependent inhibition was observed in both HLMs and RLMs, which showed reversible inhibition of CYP1A2, CYP2B1/6, CYP2C9/11, CYP2D1/6, CYP2E1 and CYP3A2/4 by plumbagin.

The present study showed that the types of inhibition by plumbagin were mixed inhibition on CYP2B6, CYP2C9, CYP2D6, CYP2E1 and CYP3A4, but non-competitive for CYP1A2 in HLMs and the *K*_i_ values were 1.62 μM, 2.16 μM, 1.46 μM, 0.65 μM, 0.88 μM and 0.15 μM, respectively. Correspondingly, mixed inhibition on CYP1A2 and CYP2D1, but competitive for CYP2B1, CYP2C11 and CYP2E1 were observed in RLMs and the *K*_i_ values were 2.64 μM, 9.49 μM, 9.93 μM, 7.85 μM, and 6.28 μM, respectively. Thus, plumbagin was a potent inhibitor on the above CYPs in both humans and rats.

Based on the inhibition results of HLMs and RLMs, some differences should be noticed such as the types of CYP inhibition and inhibitory intensity. The *K*_i_ values of inhibition on CYPs by plumbagin in HLMs are generally lower than those in RLMs. It may be attributed to variations in CYP levels, inducibilities and the existence of different CYP isoforms of the same protein family or subfamily in different species^39^. For example, CYP2C11 accounts for about 50% in total rat CYPs while human CYP2C9 being a homologue of the rat CYP2C11 with 77% homology only accounts for 20% of total human CYPs^40^. In addition, the enzyme inhibition kinetic of CYP3A2 disagreed with the classical Michaelis–Menten kinetics in RLMs, but the enzyme inhibition kinetic of CYP3A4 in HLMs conformed to that when the concentrations of midazolam were no more than 5 μΜ. The differences between CYP3A2 and CYP3A4 could also be a reflection of species differences. Consequently, extrapolating results from animals to humans should be cautious.

As mentioned above, when investigating the enzyme inhibition kinetic of CYP3A2, we found it did not conform to the classical Michaelis–Menten kinetics in RLMs. In fact, metabolic activation was achieved by incubation of plumbagin in the presence of CYP3A2 when the concentrations of midazolam are less than 5 μΜ in RLMs^41^. In this report, however, the unusual effects of plumbagin on CYP3A2 could be explained using an atypical kinetics model. Atypical Michaelis–Menten kinetics including substrate inhibition, partial inhibition, activation, auto-activation and biphasic metabolism has been demonstrated for some CYP3A-mediated reactions^42^,^43^. In this study, CYP3A2 displayed auto-activation kinetics. Multisite hypothesis was a possible mechanistic model of atypical kinetics. CYP3A enzyme except for two or more midazolam-binding sites contains an activator binding site which is closely related to the substrate binding sites^42^. Plumbagin might lead to metabolic activation by binding to the activator binding sites. These speculations still need to be further proved by experiments.

Previous pharmacokinetic study of plumbagin by oral administration (100 mg/kg) in rats showed that the peak concentration (C_max_), half-life (t_1/2_) and area under the curve (AUC) of plumbagin in the plasma was 0.35 μg/ml (1.86 μM), 1028 min and 271.9 min*μg/ml, respectively^3^. Therefore, the rat plasma concentrations were close to the IC_50_ and *K*_i_ values determined in this study, thus suggesting plumbagin could quite probably decrease the activities of CYP1A2, CYP2B1, CYP2C11, CYP2D1, CYP2E1 and CYP3A2 *in vivo*. In fact, these CYP homologues in humans are responsible for 80% of the CYP-mediated metabolism including a number of drugs with narrow therapeutic ranges. Therefore the potential HDIs should be brought to attention and further studies *in vivo* are needed to confirm the interactions of plumbagin with CYP isoforms in humans.

In conclusion, the data in this study demonstrated that plumbagin exhibited potently broad-spectrum inhibitory effects toward CYP enzymes including CYP1A2, CYP2B1/6, CYP2C9/11, CYP2D1/6, CYP2E1 and CYP3A2/4 with lower *K*_i_ values in both human and rat liver. In particular, plumbagin was not only a mixed inhibitor of CYP2B6, CYP2C9, CYP2D6, CYP2E1 and CYP3A4, but also a non-competitive inhibition of CYP1A2 in humans. These results provided useful guidance for safe and effective usage of plumbaign in clinic, especially in combination with other drugs.

## Methods

### Materials and chemicals

Pooled human liver microsomes (n = 20) were obtained from Corning Gentest Corporation (Woburn, MA, USA) and stored at −150 °C until use. All the experimental procedures involving humans have been carried out in accordance with The Code of Ethics of the World Medical Association (Declaration of Helsinki) guidelines. Phenacetin, bupropion, tolbutamide, dextromethorphan, midazolam, 3-acetamidophenol (internal standard), chlorpropamide (internal standard), glucose 6-phosphate (G6P), glucose 6-phosphate dehydrogenase (G6PDH), β-nicotinamide adenine dinucleotide phosphate (NADP) and tris (hydroxymethyl) aminomethane hydrochloride (Tris-HCl) were purchased from Sigma Chemical Co. (St. Louis, MO, USA). Chlorzoxazone was purchased from Alfa Aesar (Massachusetts, USA). 4-acetamidophenol, hydroxybupropion, 4-hydroxytolbutamide, dextrorphan, 6-hydroxychlorzoxazone and 1-hydroxymidazolam were obtained from Toronto Research Chemical (North York, Canada). Mebendazole (internal standard) was obtained from Aladdin Industrial Co. (California, USA). Plumbagin (purity > 98%) was purchased from Dalian Meilun Biotech Co., Ltd (Dalian, China). Acetonitrile and methanol (all HPLC grade) were purchased from Fisher Chemicals (Leicester, UK). Formic acid (HPLC grade) was purchased from TEDIA (Ohio, USA). Distilled water was purified in a Millipore system Milli Q (Millipore Corp., Bedford, MA, USA).

### Animals

Male Sprague-Dawley rats (200–250 g) were supplied by National Rodent Laboratory Animal Resources, Shanghai Branch of China. The animals were kept in a specific pathogen-free facility and with access to rodent cubes and sterile water, with 12 h light-dark cycles. All the methods in animals were carried out in accordance with the National Institutes of Health standards established in the ‘Guidelines for the Care and Use of Experimental Animals’. All experimental protocols in animals were approved by the Ethics Committee on Animal Experimentation of East China Normal University (Shanghai, China).

### Preparation of rat liver microsomes

In this study, the rats were fasted overnight and killed by cervical dislocation before removal of the liver. The liver was excised, rinsed with ice-cold saline (0.9% NaCl w/v), weighed and homogenized in a 0.05 mM Tris/KCl buffer (pH 7.4). Subsequently, the homogenate was centrifuged at 10,000 × *g* at 4 °C for 30 min. The supernatant was then centrifuged at 105,000 × *g* at 4 °C for 60 min. The pellet was reconstituted with 0.05 mM Tris/KCl buffer (pH 7.4) and stored at −150 °C until use. The protein concentration of the liver microsomes was determined by a protein quantitative assay using bicinchoninic acid^22^.

### Microsomal incubation

Microsomal incubation was carried out at 37 °C in a mixture of 200 μl in 0.05 M Tris/HCl buffer (pH 7.4) consisting of pooled HLMs (0.5 mg/ml) or RLMs (1 mg/ml) and an NADPH-regenerating system including MgCl_2_ (5 mM), G6P (10 mM), G6PDH (0.4 U/ml) and NADP (1 mM). Substrates were added into the incubation mixture and obtained the final concentrations (10 μM phenacetin for CYP1A2, 20 μM bupropion for CYP2B1/6, 20 μM tolbutamide for CYP2C9/11, 5 μM dextromethorphan for CYP2D1/6, 20 μM chlorzoxazone for CYP2E1 and 10 μM midazolam for CYP3A2/4), which were close to *K*_m_ respectively. The proportion of organic solvent was not higher than 1% (v/v) in the incubation mixture^18^,^44^. After pre-incubation for 5 min, the reaction was initiated by addition of NADP. Incubations were performed for 20 min, stopped by adding 200 μl ice-cold acetonitrile. And then 20 μl internal standard solution was added (3-acetamidophenol 2 μg/ml, chlorpropamide 2 μg/ml and mebendazole 2 μg/ml). The solution was mixed thoroughly for 3 min and centrifuged for 10 min at 16,000 × *g* at 4 °C. The supernatant (60 μl) was transferred to autosampler vials, and 2 μl was injected into LC-MS/MS system.

### Determination of IC_50_ values for inhibition

The IC_50_ (concentration of inhibitor to cause 50% inhibition of original enzyme activity) values were determined with the same incubation condition above. The specific concentrations of plumbagin were ranged from 0.1 μM to 50 μM. The IC_50_ values were calculated by plotting relative activities over the logarithm of plumbagin concentrations using GraphPad Prism 5.0 (GraphPad software Inc., CA, USA).

### Mechanism of inhibition of CYPs by plumbagin

To explore the time-dependent inhibition of plumbagin on CYPs, inactivation incubation containing plumbagin whose final concentration close to IC_25_ (concentration of inhibitor to cause 25% inhibition of original enzyme activity)^37^, pooled HLMs (0.5 mg/ml) or RLMs (1 mg/ml), MgCl_2_ (5 mM), G6P (10 mM), G6PDH (0.4 U/ml), NADP (1 mM) and 0.05 M Tris/HCl buffer (pH 7.4) was performed for 0, 5, 10 and 20 min at 37 °C. Cocktail probe substrates were then added and incubated for 20 min. Reactions were stopped with 200 μl ice-cold acetonitrile. The samples were treated according to the microsomal incubation method and then injected into LC-MS/MS for analysis.

Kinetic studies were performed for determining the types of inhibition on CYP by plumbagin. Pooled HLMs (0.5 mg/ml) or RLMs (1 mg/ml) with plumbagin (1 μM, 2 μM, 5 μM and 10 μM) were incubated in 0.05 M Tris/HCl buffer (pH 7.4) for 20 min at 37 °C. A series of concentrations for cocktail probe substrates were used: 2.5–20 μM phenacetin for CYP1A2; 5–40 μM bupropion for CYP2B1/6; 5–40 μM tolbutamide for CYP2C9/11; 1.25–10 μM dextromethorphan for CYP2D1/6; 5–40 μM chlorzoxazone for CYP2E1; 2.5–80 μM midazolam for CYP3A2; and 0.625–5 μM midazolam for CYP3A4. Enzyme kinetics as well as statistical analysis were performed using GraphPad Prism 5.0.

### LC-MS/MS analysis

**Figure 6:**
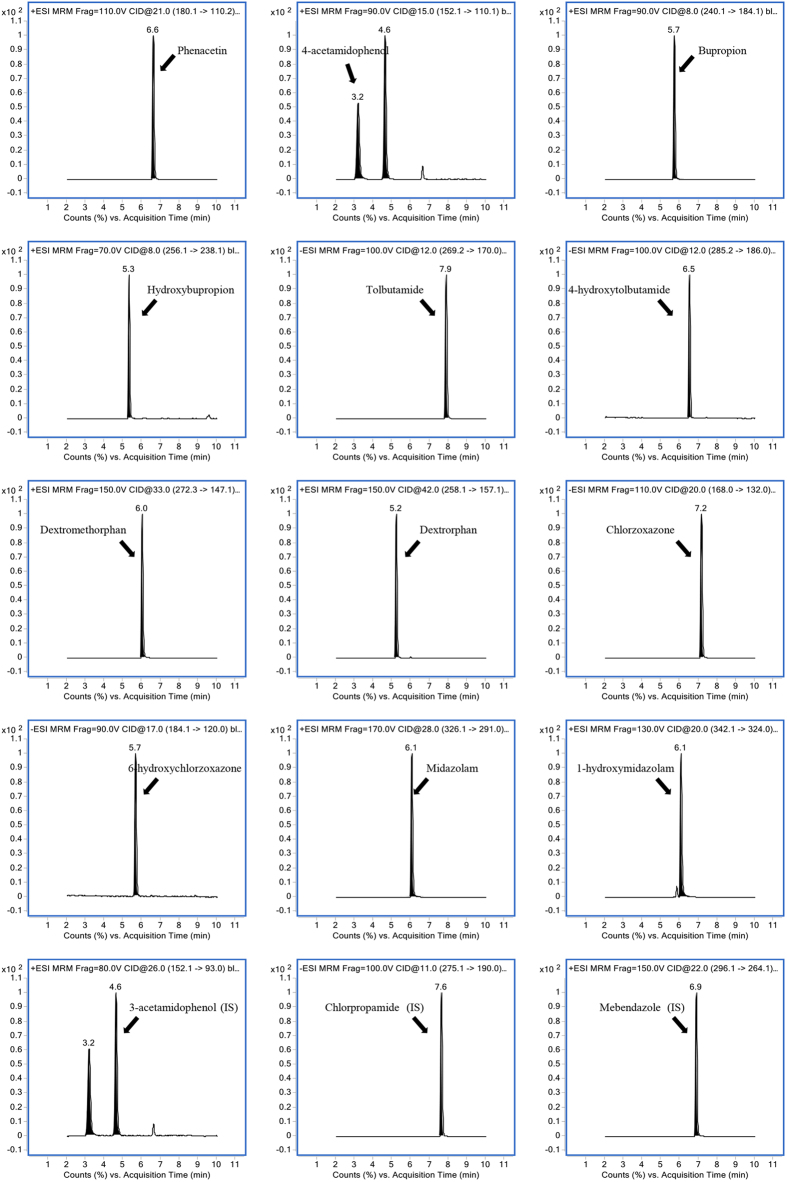
Representative multiple reaction monitoring chromatograms of CYP probe substrates, metabolites and internal standards.

LC-MS/MS is a powerful tool to detect the concentrations of several CYP substrates and metabolites in a short time, and to calculate the enzymatic reaction rate, which is used to evaluate the corresponding CYP enzyme activities. In this study, an Agilent 1290 LC system consisting of a degasser, a binary pump, an autosampler and a thermostatic column compartment was coupled with a 6460 triple-quadrupole mass spectrometer (Agilent Technologies, USA), which was equipped with an Agilent Jet Stream electrospray ionization (ESI) source and operated with Agilent MassHunter version 5.0.280.1 software (Agilent Technologies, USA). Chromatography separation was performed on a Phenomenex Kinetex XB-C18 column (100 × 3.00 mm, 2.6 μM) protected by a Phenomenex C18 guard column (Torrance, CA, USA). The mobile phase consisted of solvent A (0.1% formic acid in water) and solvent B (0.1% formic acid in acetonitrile) using gradient elution at a flow rate of 0.3 mL/min. The optimum condition for elution was as follows: 0–2.2 min, 10–11% B; 2.2–8.5 min, 11–90% B; 8.5–9 min, 90–92% B; 9–9.3 min, 92% B; 9.3–9.6 min, 92–10% B; 9.6–11.5 min, 10% B. The temperature of column oven was maintained at 30 °C, and the injection volume was 2 μL. The mass spectrometer was operated in the positive/negative ion-switching ESI mode, and the detection of the ions was performed in the multiple reaction monitoring (MRM) mode. Each MRM transition and the compound dependent parameters are presented in Table 2. MS/MS conditions were optimized as follows: gas temperature, 350 °C; gas flow, 10 L/min; nebulizer, 35 psi; sheath gas temperature, 350 °C; sheath gas flow, 11 L/min; collision gas (N_2_), 1.6 MPa. A divert valve was used to divert the eluent to waste from 0 to 2 min, 10 to 11.5 min, and to MS from 2 to 10 min. Representative multiple reaction monitoring chromatograms of analytes were shown in Fig. 6.

### Data analysis

All data were presented as mean ± SD. The IC_50_ values were fitted by non-linear regression analysis using GraphPad Prism 5.0. The kinetic parameters were obtained by fitting velocity data to kinetic models (Michaelis-Menten) using GraphPad Prism 5.0. The Lineweaver-Burk Plot (the reciprocal of reaction velocities versus the reciprocal of substrates concentrations) was used to determine the quality of fit to a specific inhibition model. The inhibition constant (*K*_i_) was obtained by a secondary plot using the slopes of the primary Lineweaver-Burk Plot (*K*_m_/*V*_max_ versus inhibitor concentrations). α*K*_i_ was obtained from a secondary plot using the y-intercepts of the Lineweaver-Burk plot (1/*V*_max_ versus inhibitor concentrations). All results were analyzed in quadruplicate. One-way analysis of variance was used to estimate the significance of differences. There was statistical significance between control and test groups if *p* < 0.05.

## Additional Information

**How to cite this article**: Chen, A. *et al*. Evaluation of the inhibition potential of plumbagin against cytochrome P450 using LC-MS/MS and cocktail approach. *Sci. Rep.* **6**, 28482; doi: 10.1038/srep28482 (2016).

## Figures and Tables

**Table 1:** Inhibitory effects of plumbagin on six CYP isoforms in HLMs and RLMs.

	CYP isoform	IC_50_ (μM)	*K*_i_ (μM)	α*K*_i_ (μM)	α	Type
Humans	1A2	7.49	0.15	0.16	0.94	non-competitive
	2B6	13.10	1.62	2.92	0.55	mixed-type
	2C9	10.94	2.16	1.46	1.48	mixed-type
	2D6	10.21	1.46	2.22	0.66	mixed-type
	2E1	6.54	0.65	2.64	0.25	mixed-type
	3A4	6.45	0.88	0.57	1.54	mixed-type
Rats	1A2	2.65	2.64	8.51	0.31	mixed-type
	2B1	5.86	9.93	‒	‒	competitive
	2C11	3.85	7.85	‒	‒	competitive
	2D1	6.17	9.49	13.79	0.69	mixed-type
	2E1	3.72	6.28	‒	‒	competitive
	3A2	8.47	‒	‒	‒	atypical kinetics

**Table 2:** Analytical parameters for the individual substrate, metabolite and internal standard (IS).

Analyte	Ionization mode	Precursor ion (m/z)	Product ion (m/z)	Fragmentor (V)	Collision energy (eV)
Phenacetin	ESI+	180.1	110.2	110	21
4-acetamidophenol	ESI+	152.1	110.1	90	15
Bupropion	ESI+	240.1	184.1	90	8
Hydroxybupropion	ESI+	256.1	238.1	70	8
Tolbutamide	ESI−	269.2	170.0	100	12
4-hydroxytolbutamide	ESI−	285.2	186.0	100	12
Dextromethorphan	ESI+	272.3	147.1	150	33
Dextrorphan	ESI+	258.1	157.1	150	42
Chlorzoxazone	ESI−	168.0	132.0	110	20
6-hydroxychlorzoxazone	ESI−	184.1	120.0	90	17
Midazolam	ESI+	326.1	291.0	170	28
1-hydroxymidazolam	ESI+	342.1	324.0	130	20
3-acetamidophenol (IS)	ESI+	152.1	93.0	80	26
Chlorpropamide (IS)	ESI−	275.1	190	100	11
Mebendazole (IS)	ESI+	296.1	264.1	150	22
